# New insights into *PSAT1* as a therapeutic target for myelodysplastic syndrome (MDS)

**DOI:** 10.1371/journal.pone.0309456

**Published:** 2024-08-26

**Authors:** Sael Alatawi, Waseem Alzamzami

**Affiliations:** Department of Medical Laboratory Technology, Faculty of Applied Medical Sciences, University of Tabuk, Tabuk, Saudi Arabia; UC Los Angeles: University of California Los Angeles, UNITED STATES OF AMERICA

## Abstract

The metabolomic landscape in myelodysplastic syndrome (MDS) is highly deregulated and presents promising avenues for understanding disease pathogenesis and potential molecular dependencies. Here, we evaluated the transcriptomic landscape in MDS in multiple independent studies focusing more on metabolomics pathways. Identifying molecular dependencies will pave the way for a more precise disease stratification as well as the development of novel personalized treatment strategies. The study adopted a retrospective, cross-sectional approach, utilizing transcriptomic data from multiple MDS studies. The transcriptomic data were then subjected to comprehensive analyses, including differential gene expression, gene enrichment analysis, gene co-expression analysis, protein-protein interaction analyses, and survival analyses. *PSAT1* showed a significant upregulation profile in MDS patients. This observed upregulation is correlated with the deregulation of immune-related pathways in MDS samples. This observation suggests a novel role for PSAT1 in immune modulation and potentially in augmenting immune evasion, which may lead to poor prognosis. This was evident in other tumors in the TCGA database, where cancer patients with high *PSAT1* expression have a shorter overall survival. This study unveils a novel potential therapeutic avenue in MDS. Identifying the role of the *PSAT1* gene sheds light on the disease’s intricate biology, highlighting the ongoing cross-talk between metabolism and immune regulation, which may pave the way for innovative treatment modalities.

## Introduction

Myelodysplastic syndrome (MDS) encompasses a diverse group of clonal diseases involving hematopoietic stem cells, carrying an elevated risk of progressing to acute myeloid leukemia (AML) [1]. While MDS is diagnosed in over 10,000 individuals annually in the United States, predominantly affecting older adults, it is relatively uncommon in children (2–7%). MDS progression to AML is a known complication of this disease and can lead to fatal outcomes [2–4]. Tumorigenic cells rely more heavily on rewiring the metabolome landscape to maintain sufficient energy resources and, eventually, survival [5]. This is seen in utilizing glutamine as an alternative to glucose metabolism, such as reprogramming metabolic processes toward glutamine metabolic pathways [6, 7]. Such a significant metabolic shift adds more vigor to cancer cells, leading to an active form of disease.

Although MDS patients potentially transform into AML, understanding the intricate molecular changes during MDS proliferation, especially its metabolic processes, is a vital elucidation for understanding MDS pathogenesis. Given the intricate nature of MDS pathogenesis, where the primary metabolic pathways are oxidative phosphorylation and glycolysis, serine is a pivotal oncometabolite that undergoes distinct metabolic alterations to drive cell growth and proliferation [8, 9]. Consequently, glucose and glutamine serve as primary sources utilizing essential metabolic pathways. Moreover, alterations in mitochondrial structure and genetic variations in mitochondrial proteins are critical features of MDS, yet a comprehensive understanding of the metabolic attributes and the cells responsible for energy production in MDS remains elusive [10]. Furthermore, studies in genetically modified mice with an MDS phenotype reveal a significant metabolic shift in hematopoietic stem cells (HSCs). Regardless, it is crucial to emphasize that the MDS pathophysiology is substantially affected by disturbances in the epigenome, along with multiple genetic and epigenetic abnormalities [3]. This phenotype presents an intriguing field of research.

In our comprehensive interdisciplinary investigation, we strongly emphasized the transcriptomic profile of MDS patients. We aimed to identify the novel signature genes and thoroughly evaluate molecular pathways involved in MDS pathogenesis. This comprehensive approach allowed us to identify numerous signature genes and gain profound insights into MDS biology. Our previous multi-cohort analysis work reveals the interplay between metabolism, methylation, and demethylation in MDS as potential clinical applications, the possibility of improving therapeutic avenues for MDS patients, and a foundation for prospective clinical trials and investigations. This study, meticulously designed to investigate the potential association between several molecular pathways and tumorigenesis in MDS, recognizes the disease’s inherent heterogeneity. Indeed, our findings underscore the crucial need for innovative therapeutic strategies to address the diverse spectrum of challenges in MDS.

## Methodology

### Ethics approval

Given the nature of this design, there is no need for additional ethical approval. Thus, the Local Ethics Committee waived the ethical approval. The current study investigated the publicly available data using data from three independent cohorts (GSE114922 in 2015, GSE63569 in 2018, and GSE183328 in 2022). Accordingly, a flowchart that outlines the data processing steps involved in generating the results in this study is illustrated in S1 Fig.

### Patient cohorts

This research utilized a retrospective, cross-sectional study approach, utilizing transcriptomic data from multiple datasets and studies involving patients with MDS. Inclusion criteria for this study include common cellular starting material (CD34), samples from MDS patients with no prior treatment, and a common NGS sequencing platform to minimize any potential technical noise. Three MDS studies were found to have met these criteria, and their transcriptomic data was found in public databases (GEO). Transcriptomic raw data were retrieved from the GSE database for all three studies (n = 164). These studies are GSE114922 from the Wellcome Trust Centre for Human Genetics, United Kingdom; GSE63569 from the University of Oxford, United Kingdom; and GSE183328 from CIMA, Spain, collected in 2015, 2018, and 2022, respectively [11–14]. Detailed clinical information is summarized in the supplements table (S1 and S2 Tables). Data was accessed in the NCBI GEO database on September 10, 2023, using SRA tools. The authors do not have access to information that could identify individual participants during or after data collection, as the data was already coded in the GEO database.

### Raw data processing and mapping

Raw sequencing data were QC evaluated and then mapped to *hg38* using the HISTA2 tool [15]. The obtained bam files were filtered and subjected to duplicate removal using Picard. Next, the Gene expression count matrix was obtained using the tool featureCounts [16] using the latest GTF file obtained from Gencode [17]. The resulting count matrix was then used as input for differential gene expression.

### Differential gene expression

We utilized DESeq2 [18] to identify differentially expressed genes (DEGs) between healthy individuals and those with MDS. To narrow our scope of analysis and to monitor the expression of genes with roles in metabolism in MDS versus healthy individuals, a set of genes was obtained from the Gene Ontology (GO) database (term GO:0008152) that is associated with the metabolic process in *Homo sapiens* [19, 20]. A gene was considered significant if it had an adjusted *p*-value below 0.05 and a log2 fold change of more than 1.5. Applying this stringent cut-off resulted in lists of differentially expressed genes that were upregulated and downregulated in MDS patients. Following this, Gene Set Enrichment Analysis (GSEA) [21, 22] was conducted on the differentially expressed genes, resulting in significant hallmarks. Next, healthy samples were removed from the analysis, and we then labeled MDS samples to be (High or Low) according to the median value of *PSAT1* gene expression. After this labeling, we conducted a second differential gene expression analysis by contrasting MDS samples labeled with High *PSAT1* against MDS samples labeled with Low *PSAT1*. The resulting differential expressed genes were then subjected to GSEA. All plots were generated using R.

### Gene co-expression analysis

Normalized count matrices were obtained using DESEQ2 from contrasting *PSAT1* High vs Low, and then log2 was transformed and filtered for variance. This preprocessed count matrix was then used as input for our gene-coexpression analysis. We conducted our gene-coexpression analysis using CEMiTool [23]. This analysis resulted in a set of significant modules, each with a list of genes showing a co-expression pattern. We selected the highly significant module (M1) for our downstream analysis. Module 1 (M1) genes were then subjected to functional enrichment analysis using the GO database. In addition, to evaluate the potential protein-protein interactions of genes in M1, the STRING database [24] was utilized, and a protein-protein interaction network was generated.

### Survival analysis

To assess the impact of *PSAT1* gene expression in other tumors, we screened the TCGA cohort’s transcriptomic data and associated clinical information. Normalized gene expression metrics were downloaded for each cohort from the UCSC Xena project [25], and cancer samples were labeled as High or Low according to each cohort’s median expression value of *PSAT1*. We then constructed a survival curve using the survminer package. Survival curves were considered significant if the log-rank test *p*-value was below 0.05.

## Results

### Identified MDS-derived signature genes involved in metabolic processes

**Fig 1:**
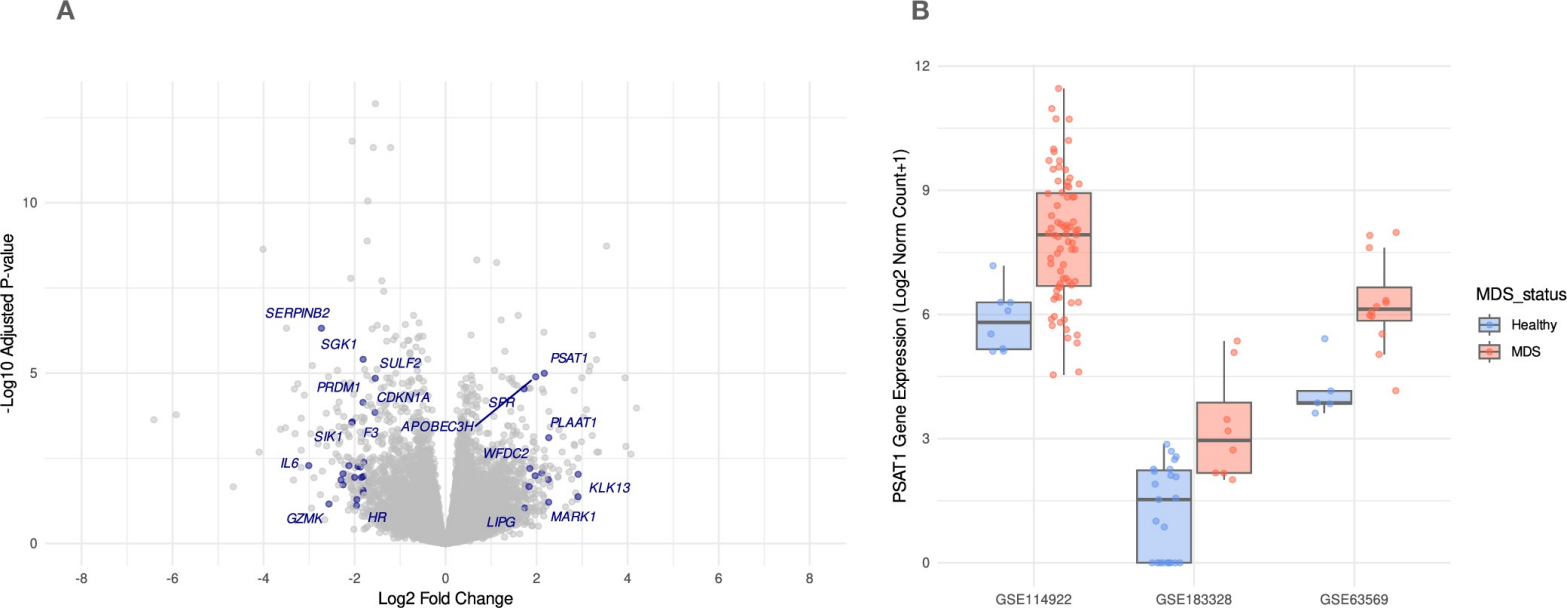
Genes with roles in metabolic processes show a deregulated gene expression profile. (A) Volcano plot showing the gene expression of significantly up/down-regulated genes in MDS cells by emphasizing a set of selected genes related to cellular metabolism, GO:0008152. (B) Box plots show the upregulation of the *PSAT1* gene in MDS individuals compared to healthy individuals among independent multiple MDS cohorts. Only genes that passed the preset significance threshold were highlighted and labeled (FDR < 0.1 and fold change -/+ 1.5).

The metabolic landscape in MDS is highly deregulated and driven by pathways that possess a disease vulnerability. To narrow our scope of analysis within this context, we identified signature genes associated with the metabolic process by employing a particular GO term, explicitly utilizing the GO term, GO:0008152, for this identification [19, 20]. These genes, which we monitored for their expression in MDS compared to healthy individuals, showed a significant gene expression profile, as represented in, (Fig 1A). Our analysis pinpointed the serpin family B member 2 (*SERPINB2*) and serum/glucocorticoid regulated kinase 1 (*SGK1*) genes as the most downregulated gene in MDS cells, while the phosphoserine aminotransferase 1 (*PSAT1*) gene and sepiapterin reductase (*SPR*) genes were the most upregulated in MDS cells. These findings underscore the significance of these signature genes in the context of MDS.

Then, our analysis found that *PSAT1* gene expression was upregulated in each independent MDS cohort (Fig 1B), suggesting metabolic dependency in MDS cells. As to our knowledge, the role of *PSAT1* has not been reported in MDS, so these observations have prompted us to speculate about its role in this hematological malignancy. These findings highlight the potential importance of *PSAT1* in MDS pathogenesis and require further investigation into its mechanistic role and therapeutic implications in this disease.

### *PSAT1* drives the gene expression landscape into metabolic-immune pathways in MDS

To better understand the impact of *PSAT1* on the overall transcriptomic profile in MDS, we removed healthy samples from the analysis and labeled MDS samples as high or low based on the median gene expression of *PSAT1*. Next, we performed a second DEG analysis by contrasting MDS samples with high *PSAT1* gene expression against samples with low expression. Using this approach will help to obtain insights into what molecular pathways are correlated with high *PSAT1* and vice versa. Then, a list of statistically significant genes was obtained from this analysis.

**Fig 2:**
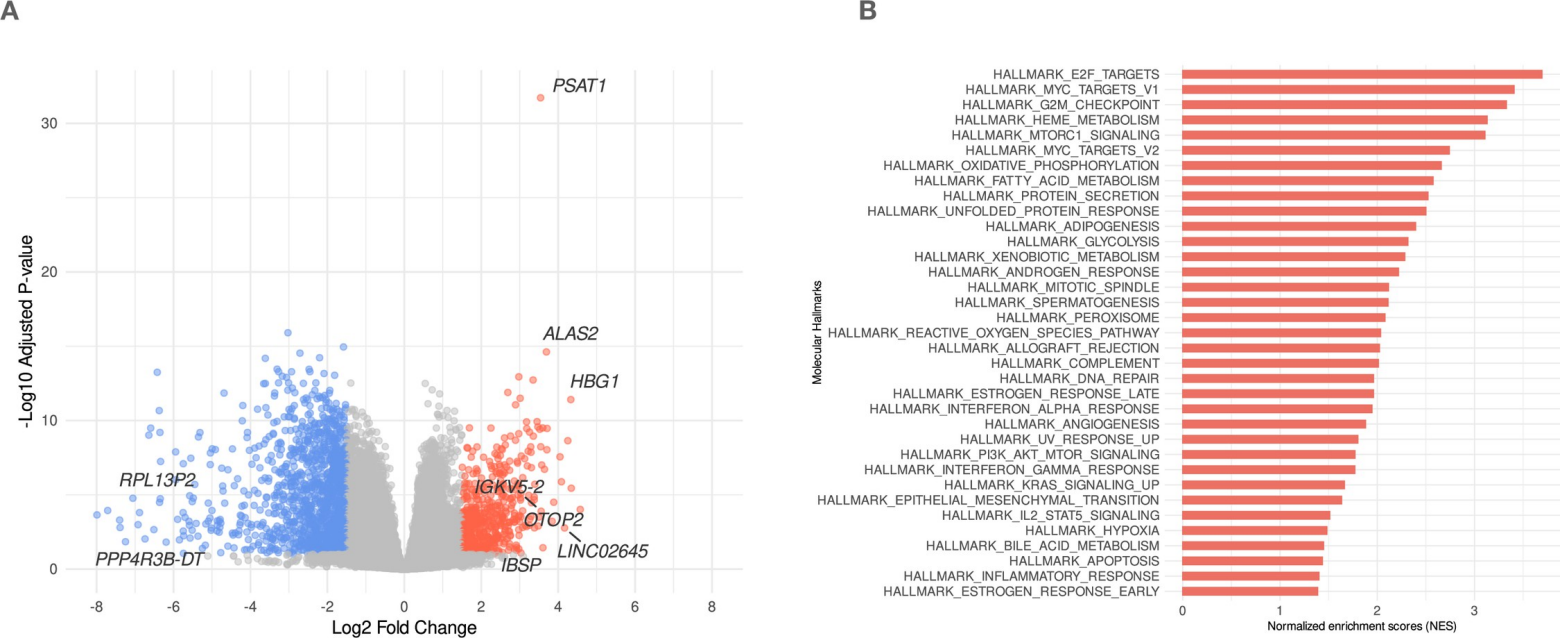
*PSAT1* upregulation is correlated with alterations in metabolic-immune pathways. (A) Volcano plot showing the topmost significantly upregulated gene in MDS cells (*PSAT1* high vs low). (B) GSEA hallmark pathways depict the topmost enriched gene sets’ normalized enrichment scores (NES) values. Only genes that passed the preset significance threshold were highlighted and labeled (FDR < 0.1 and fold change -/+ 1.5).

Furthermore, by applying this approach, we identified that the *PSAT1* gene was among the topmost upregulated genes (which serves as a positive control) in the MDS samples labeled with *PSAT1* high, as shown in (Fig 2A) for this analysis. Next, to gain molecular insights into this list of differentially expressed genes, we conducted a GSEA analysis. We identified that most of the topmost gene sets were hallmarks in metabolic-immune pathways, as shown in (Fig 2B). These results validate the upregulation of *PSAT1* in MDS and suggest that the alteration of metabolic-immune pathways could be central to the disease’s progression and pathophysiology. However, the mechanism behind these observed alterations in metabolic-immune pathways is unknown.

**Fig 3:**
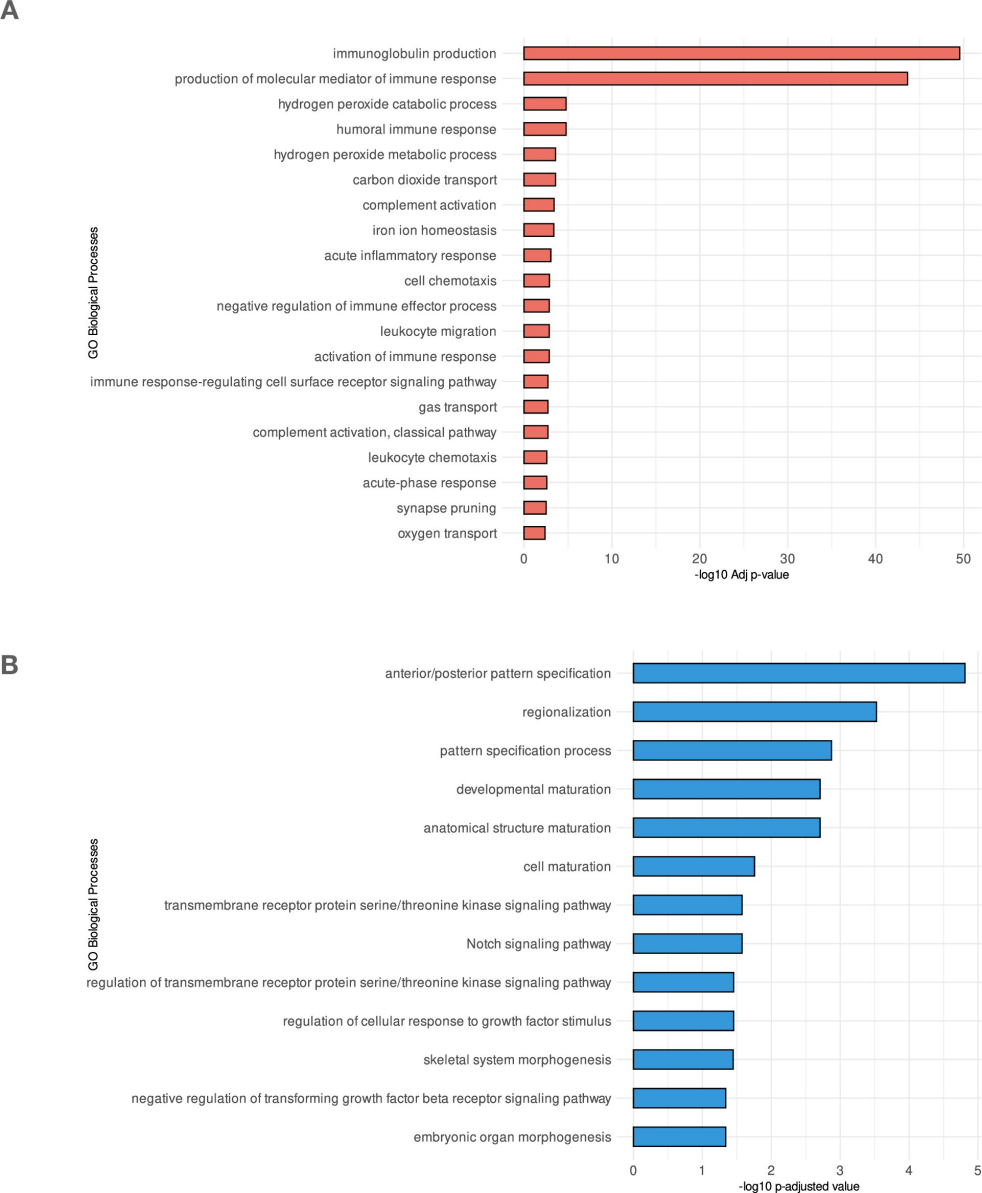
Gene functional enrichment analysis of top hallmark pathways in *PSAT1* high vs. low individual condition. (A) GO pathways resulted from downregulated genes ranked by their significance (adjusted *p*-value) (B) GO pathways resulted from upregulated genes ranked by their significance (adjusted *p*-value).

Afterward, to better understand the functional roles of differentially expressed genes assigned with up and down-regulation, we subsetted upregulated and downregulated genes based on the cut-off we selected (LogFC > 1.5 and *p* adj < 0.05). The upregulated genes showed similar results to our previous GSEA analysis (Fig 3A), where immune-related pathways were highly enriched. In contrast, the downregulated genes showed no enrichment of metabolic-immune pathways, as shown in (Fig 3B) suggesting that the gene expression landscape is mainly oriented toward metabolic-immune pathways. This disparity in pathway enrichment between upregulated and downregulated genes underscores the specific impact of metabolic-immune alterations on the upregulated gene expression profile in MDS, pointing to a gene expression program driven by *PSAT1*.

### Gene co-expression analysis identified a core network of protein-protein interaction governed by PSAT1 in MDS

**Fig 4:**
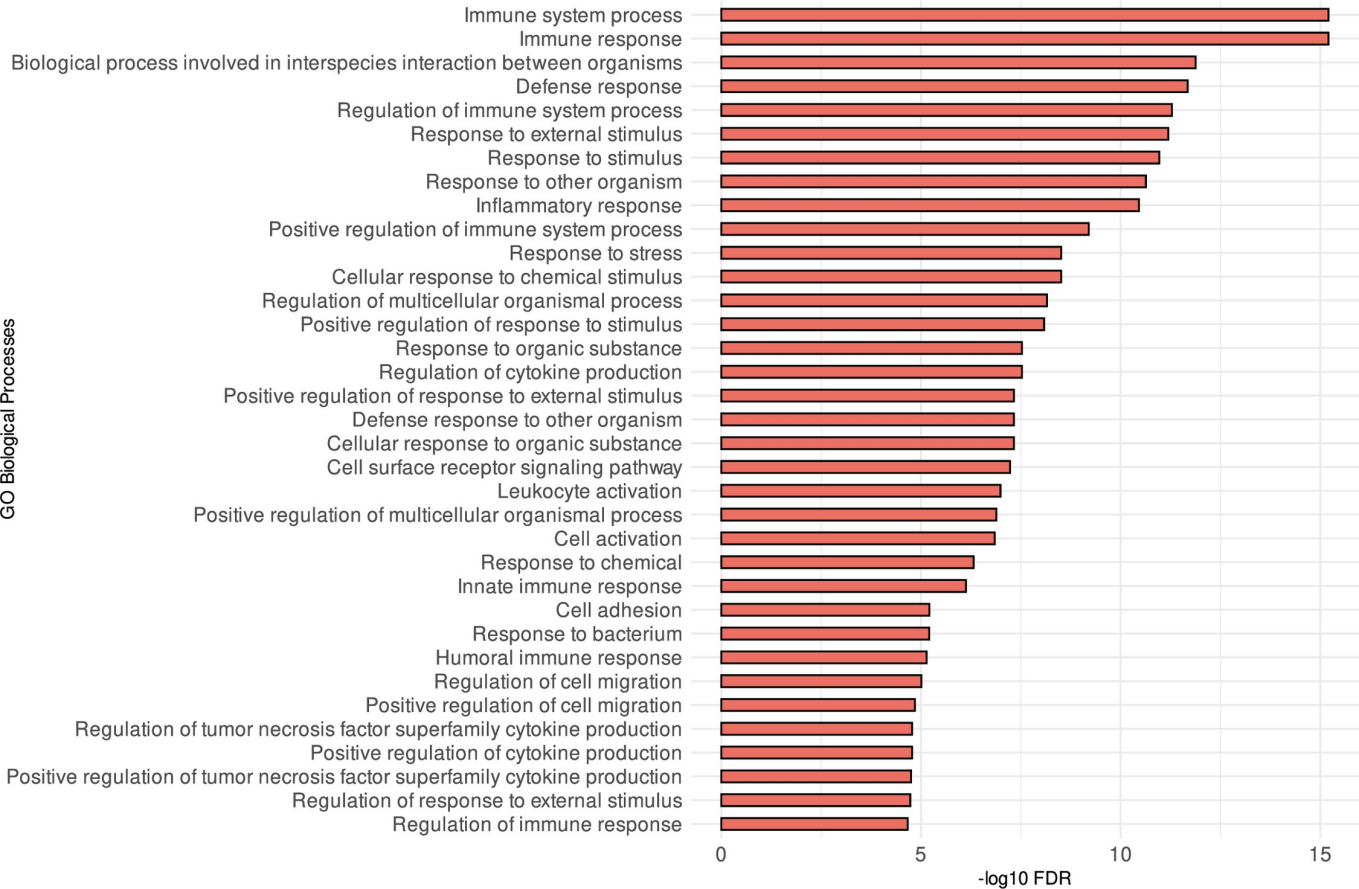
Co-expressed genes in M1 show enrichments in immune-related pathways. GO pathways resulted from the M1 gene set ranked by their significance (adjusted *p*-value).

We next turned our investigation to conduct gene co-expression analysis to gain holistic insights into mechanisms driving the gene expression program in MDS samples with marked *PSAT1* high and low. The co-expression analysis identified sets of modules with groups of genes that show a co-expression pattern. Among those modules, a significant module was identified as M1, shown in (S3 Table). This module contains a set of genes shown to have patterns of gene expression across *PSAT1* high and low samples. We performed a functional enrichment analysis to understand the common biological nature of genes in the M1 module. The results highlighted the oncometabolite and/or immune-related pathways, as evidenced by our GO functional enrichment analysis (Fig 4), which is in agreement with our previous analyses.

**Fig 5:**
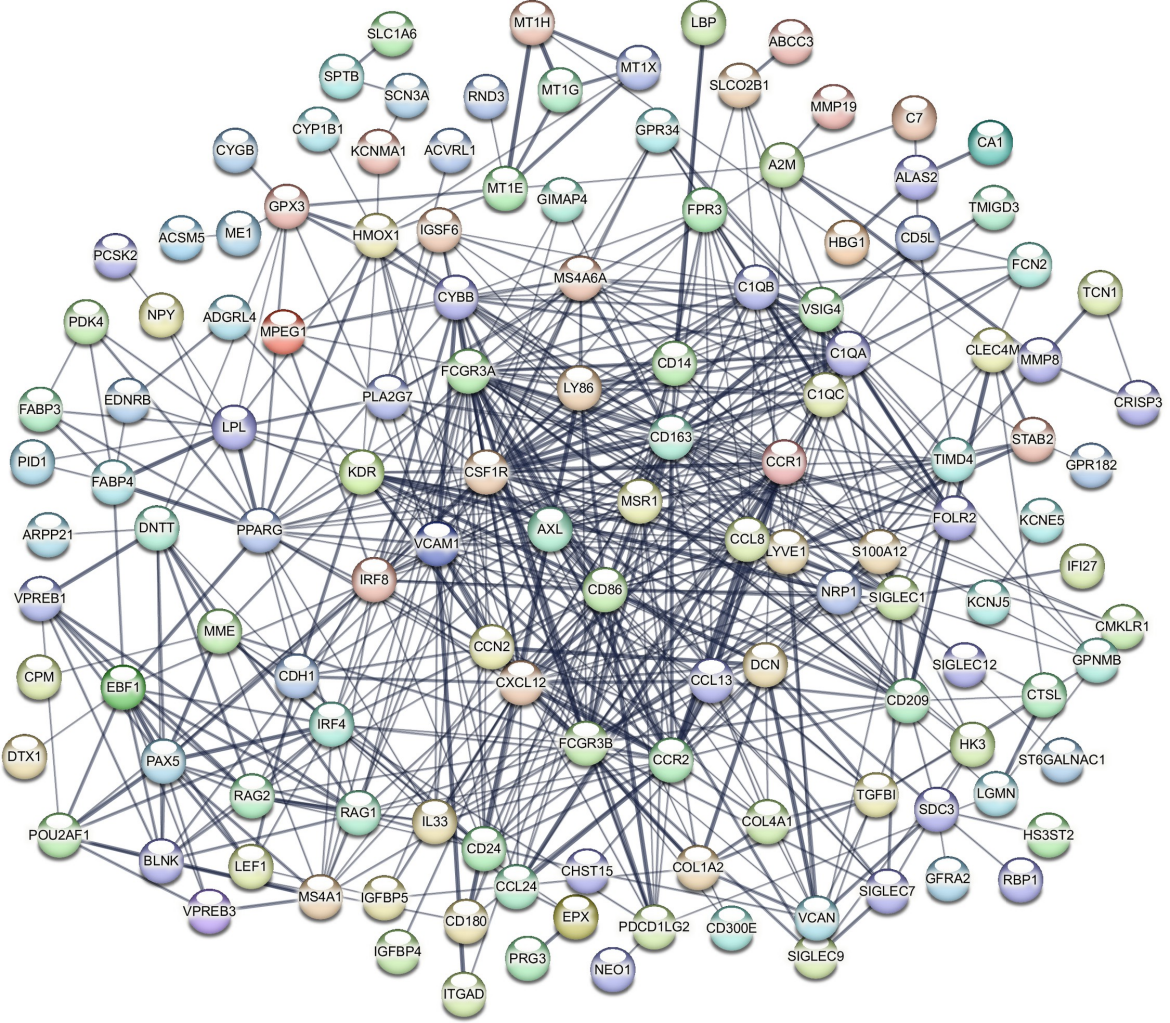
STRING network of co-expressed genes in M1 show enrichments in immune-related pathways. A STRING network of protein-protein interactions shows the biochemical reactions delivered in immune-related pathways.

Then, we conducted a protein-protein interaction analysis using the STRING database to gain further insights into the role of the genes enriched in the M1 module at the protein level. This analysis identified a significant network of protein interactions, with most proteins sharing roles in immune-related pathways, as shown in Fig 5. Of note, peroxisome proliferator-activated receptor Gamma (PPARG), C-C motif chemokine receptor 1 (CCR1), Fc gamma receptor IIIa (FCGR3A), CD163 molecule (CD163), colony-stimulating factor 1 receptor (CSF1R), and CD86 molecule (CD86) proteins showed high interaction levels. Together, these results suggest that PSAT1 is a dominant immune regulator in MDS.

### *PSAT1* increased the pathogenic regulation of immune-related pathways in MDS patients

**Fig 6:**
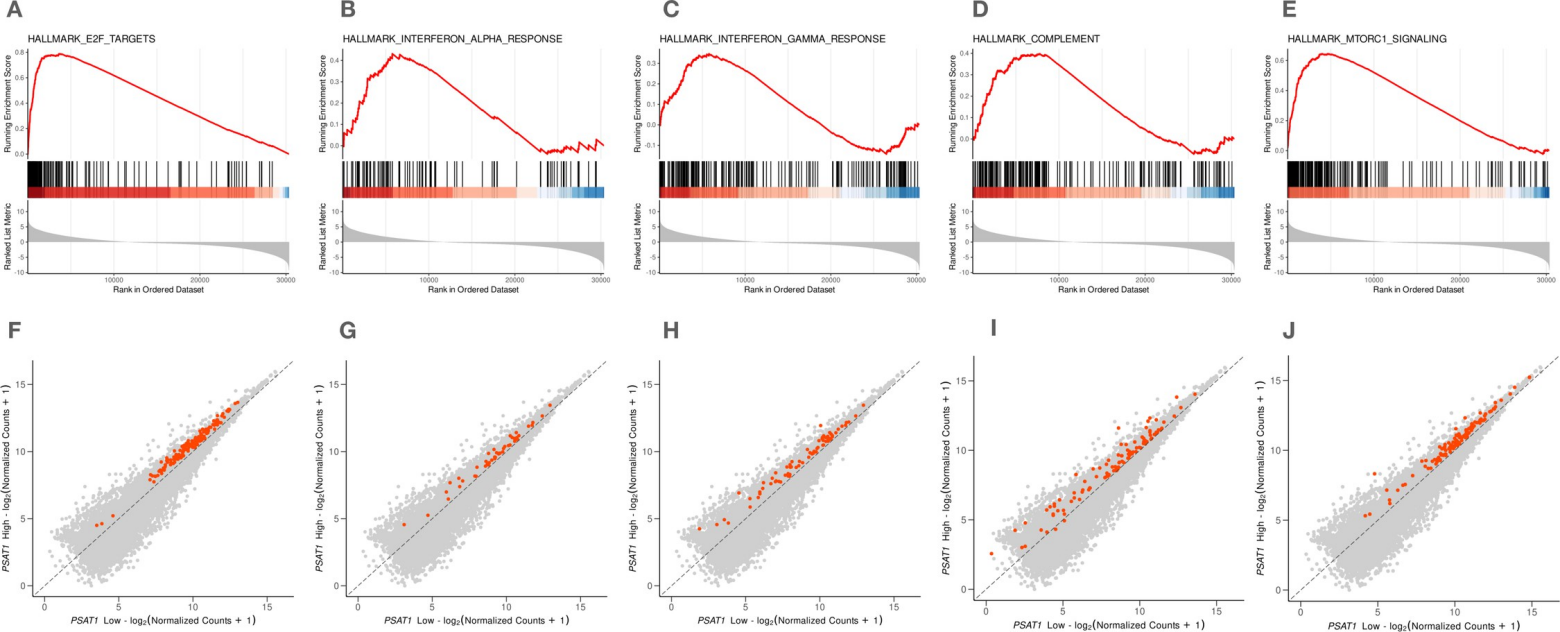
High *PSAT1* gene expression is correlated with enrichments in immune-related pathways. E2F targets, interferon α and γ, complement system components, and mTORC1 hallmarks were identified as being enriched and showed a strong correlation with elevated *PSAT1* expression levels. (A-E) Gene set enrichment analysis (GSEA) was performed on differentially expressed genes associated with *PSAT1* gene expression (High vs. Low). This analysis resulted in a set of significant hallmark signatures associated with the immune system. Hallmarks with a false discovery rate (FDR) below 0.05 were considered significant. (F-J) Genes associated with each previous hallmark were assessed in terms of gene expression further to evaluate their contribution to the aforementioned significant hallmarks. Scatter plots showed, indeed, the upregulation of immune-related genes with MDS samples with high *PSAT1* gene expression.

Given the potential immune modulation role of *PSAT1* in MDS cells, we further wanted to evaluate the expression of genes involved in immune-related pathways. Differentially expressed genes resulting from contrasting the *PSAT1* high vs. low showed a list of significant GSEA hallmarks. We identified E2F targets, interferon α, interferon γ, and complement, mTORC1 hallmarks, which were enriched and correlated with high *PSAT1*, as shown in Fig 6. We also showed that the individual gene expression of gene members of these hallmarks was indeed upregulated and orientated toward high *PSAT1*. These data suggested that high *PSAT1* gene expression is associated with activated immune-related pathways in MDS. Also, the findings emphasize the significant importance of immune modulation in the MDS pathogenesis role by *PSAT1* and indicate its potential role as a target for therapeutic strategies, enhancing our comprehension of the underlying pathophysiology of the disease.

### *PSAT1* upregulation predicts patients’ survival in cancer

**Fig 7:**
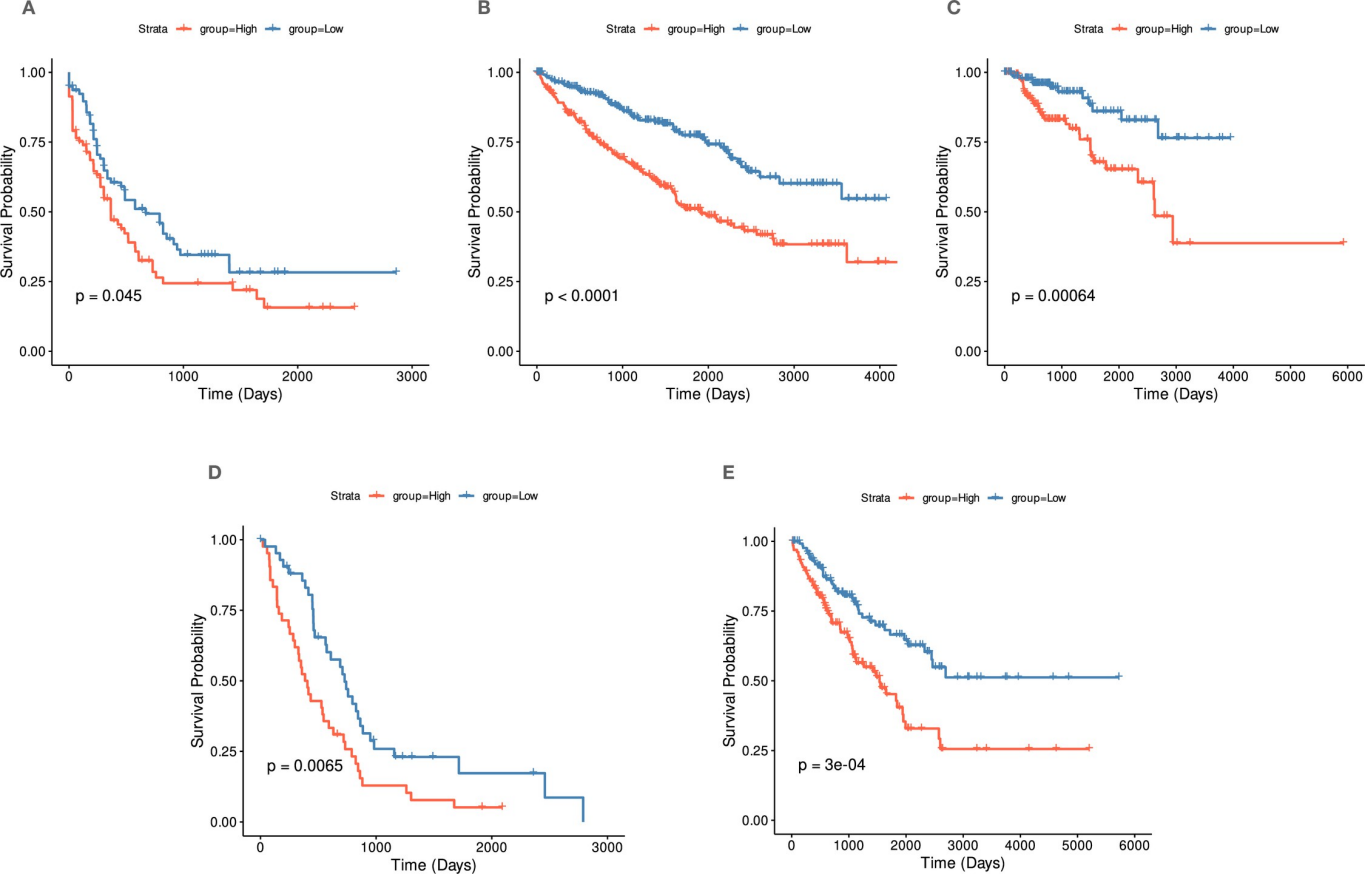
Prognostic prediction of *PSAT1* high vs. low genes in multiple independent cohorts. (A) Overall, the Survival plot by Kaplan-Meier analysis shows the difference between the high-risk and low-risk patient groups in LAML. (B) Overall, the Survival plot by Kaplan-Meier analysis shows the difference between the high-risk and low-risk patient groups in KIRC. (C) Overall, the Survival plot by Kaplan-Meier analysis shows the difference between the high-risk and low-risk patient groups in KIRP. (D). Overall, the Survival plot by Kaplan-Meier analysis shows the difference between the high-risk and low-risk patient groups in MESO. (E) Overall, the Survival plot by Kaplan-Meier analysis shows the difference between the high-risk and low-risk patient groups in SARC. The *P*-values were indicated for each cohort. Red represented patients who were classified as high-risk, and blue represented patients classified as low-risk.

We subsequently decided to predict the survival of patients across multiple independent, well-characterized public cohorts based on *PSAT1* gene expression. We screened the TCGA database for the impact of *PSAT1* gene expression on survival in more than 30 cohorts. The survival analysis was applied to develop survival prognostic scores among the independent cohorts using Kaplan-Meier curves (Fig 7). This analysis resulted in significant results in cohorts LAML (Acute Myeloid Leukemia), KIRC (Kidney Renal Clear Cell Carcinoma), KIRP (Kidney renal papillary cell carcinoma), MESO (Mesothelioma) and SARC (Sarcoma).

In the LAML cohort, the probability of the overall survival (OS) analysis showed that patients labeled with a high *PSAT1* gene expression showed a reduction in overall survival compared to patients with low *PSAT1* gene expression (*p*-value = 0.045) (Fig 7A). Cohorts KIRC, KIRP, MESO, and SARC showed similar patterns of a significant reduction in patient survival when they have a high *PSAT1* gene expression (*p*-value < 0.0001), (*p*-value = 0.00064), (*p*-value = 0.0065), and (*p*-value = 0.3E-04), respectively (Fig 7B–7E). These results suggest that the *PSAT1* high expression group of patients had a significantly poor prognosis compared to those with *PSAT1* low expression group patients. In LAML and MESO cohorts, *PSAT1* high expression group patients had a significantly poor prognosis associated with a relatively short (< 5 years) compared to the patients in *PSAT1* low expression group patients. These findings emphasize the prognostic significance of *PSAT1* expression across multiple cancer types, reinforcing its potential as a biomarker for predicting patient survival and guiding therapeutic strategies.

## Discussion

Cancer is a multifaceted disease influenced by various mechanisms across genomic, transcriptional, translational, biochemical, and metabolic layers. Systems biology methods allow for analyzing complex interactions within and between cancer cells’ internal and external environments and their genetic and epigenetic frameworks [26]. In this research, we utilized systems biology to explore gene co-expression networks potentially linked to immune rewiring in MDS. The upregulation of inflammatory pathways that resulted from several lines of analyses is a known driver of MDS pathogenesis [27]. Additionally, this work integrated independent MDS cohort transcriptomic datasets and then monitored the metabolome’s expression profile across all samples. Among genes used to monitor the expression profile of the metabolome is *PSAT1*, which was significantly upregulated. This gene showed a variable gene expression profile among MDS samples. Given this heterogeneous expression profile, no research on *PSAT1* has been reported in the MDS context, and the recent pieces of evidence of *PSAT1* involvement in cancer [28–30] motivated us to explore its impact in this study.

One key player manipulating cancer cells’ metabolic pathways is *PSAT1* [31]. PSAT1 is a class V aminotransferase family member and is a crucial rate-limiting enzyme in the serine-glycine synthesis pathway. Glycine, a vital nutrient for the proliferation of malignant cells, is produced in this pathway. Specifically, PSAT1 converts 3-phosphohydroxypyruvate to L-phosphoserine through a glutamate-linked transamination reaction, marking the second step in the serine-glycine biosynthesis pathway [32]. PSAT1 also orchestrates several other vital metabolic pathways, including glycolysis, *de novo* the citric acid cycle, and one-carbon metabolism, which are essential for cell proliferation, survival, migration, and epigenetic processes [19, 20, 33]. However, to our knowledge, no previous studies have been reported or linked the involvement of the *PSAT1* gene in the context of the MDS as a player in metabolome and immune rewiring. This unique aspect of research, combined with the growing evidence of *PSAT1* oncogenic regulation in solid tumor pathogenesis and metastasis [34, 35], suggests that *PSAT1* might play a potential role in the immune modulation in MDS cells.

In our analysis, we contrasted the gene expression of MDS samples labeled with high *PSAT1* against samples with low *PSAT1*. As a result of this comparison, we observed an enrichment of immune-related pathways exclusively in upregulated genes (Fig 3B). This observation agrees with recent research that shows the involvement of *PSAT1* in immune infiltration in non-small cell lung cancer (NSCLC) [29]. Our gene co-expression analysis identified patterns of co-expressed genes in MDS samples categorized based on *PSAT1* gene expression. The genes identified in the co-expression module showed high enrichment in molecular pathways related to the immune system (Fig 4). Indeed, Fig 5 shows dominant proteins sharing roles in immune-related pathways such as PPARG, CCR1, FCGR3A, CD163, CSF1R, and CD86 proteins. Thus, the heightened expression of *PSAT1* emerges as a central immunoregulatory factor in the context of MDS.

Serine is a crucial immune metabolite that significantly influences adaptive immunity by regulating the proliferative capacity of T cells [36]. In addition, the mammalian target of rapamycin (mTOR), which was enriched in results (Fig 6E), is a serine-threonine kinase that regulates cellular growth, division, and differentiation by coordinating the availability of nutrients and growth factors [37]. mTOR enhances serine synthesis and metabolism by augmenting the activity of critical enzymes [38]. The mTOR influences the growth of T and B cells by promoting their development and maturation [37, 39]. Furthermore, treatments that inhibit mTOR have proven effective in treating B-cell malignancies, highlighting the pivotal role of this pathway in cancer cell survival [40]. Together, these findings provide key evidence linking serine regulation by *PSAT1* metabolism to antitumor immunity in cancer.

Our findings link the high expression of *PSAT1* in the MDS to immune regulation. Herein, we observed extensive enrichments of molecular hallmarks such as E2F targets, interferon α, interferon γ, and complement across multiple independent MDS cohorts, as depicted in (Fig 6A–6D) Moreover, we observed gene sets constituting these molecular hallmarks are upregulated and correlate with high *PSAT1* gene expression (Fig 6F–6I). These observations suggest that gene members of these molecular hallmarks are indeed upregulated, providing support evidence for these enriched molecular hallmarks. Our results show that the consistent observed phenotypes in immune deregulation among MDS-independent cohorts were most likely attributed to the *PSAT1*.

Definitively, applying our findings to the clinical context will help to evaluate the impact of *PSAT1* gene expression on disease outcomes. We screened the gene transcriptomic data sets and associated clinical information using well-characterized cancer cohorts in the TCGA project to achieve this. The survival analysis results demonstrated that high *PSAT1* gene expression is correlated with short overall survival and possibly a poor disease prognosis in cohorts LAML, KIRC, MESO, KIRP, and SARC. Our investigation into TCGA cohorts revealed a consistent pattern of *PSAT1* expression influencing disease outcomes, which is in parallel with previous reports [28, 30, 41]. This consistency across diverse cohorts underscores the robustness of our findings and the potential of *PSAT1’s* global role in oncogenesis.

Finally, an in-depth analysis of these data could provide further insights into the molecular mechanisms by which *PSAT1* influences metabolic and immune pathways in cancer. Our analysis suggests a potential *PSAT1*-mediated crosstalk between metabolism and the immune system. Further functional studies are necessary to elucidate the specific roles of *PSAT1* in immune regulation within MDS.

## Conclusion

Our objective was to identify novel signature genes and comprehensively assess the molecular pathways implicated in MDS pathogenesis. This study not only elucidates the regulatory role of *PSAT1* in immune pathways within MDS but also presents potential clinical applications. This pattern drives the expression of the *PSAT1* gene to be highly attributed to and involved in immunoediting as a player in MDS pathogenesis. These insights into enhancing therapeutic options for MDS patients are particularly relevant, as they could significantly impact patient care. Furthermore, this research lays a solid foundation for prospective clinical trials and further investigations, underscoring its importance in advancing our understanding of MDS.

## Supporting information

S1 Fig A flowchart that outlines the data processing steps involved in the study. (TIF)

S1 Table Clinical characteristics of the three independent cohorts. (XLSX)

S2 Table Disease subtype of each patient among the three independent cohorts. (XLSX)

S3 Table Modules correspondent gene lists derived from gene co-expression analysis. (XLSX)
