# Supplementary figures and images for: New insights into PSAT1 as a therapeutic target for myelodysplastic syndrome (MDS)
Source: PLoS One. 2024 Aug 26;19(8):e0309456. doi: 10.1371/journal.pone.0309456 (PMC11346733; doi:10.1371/journal.pone.0309456)

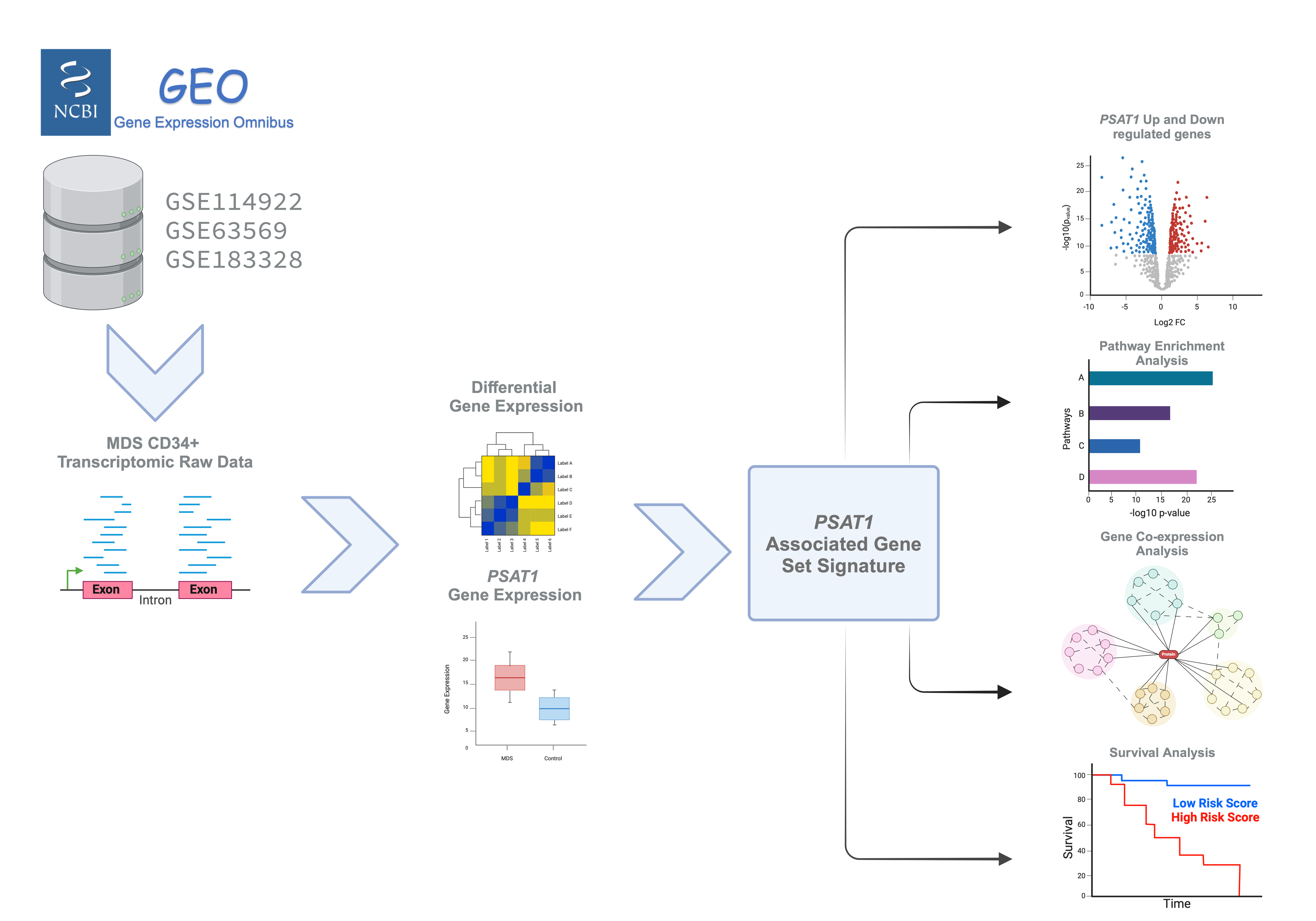

Supplement: S1 Fig — (TIF) [file pone.0309456.s001.tif]
